# Biologics for Inflammatory Bowel Disease in Clinical Practice: A Calabria (Southern Italy) Prospective Pharmacovigilance Study

**DOI:** 10.3390/pharmaceutics14112449

**Published:** 2022-11-13

**Authors:** Martina Tallarico, Caterina Palleria, Livia Ruffolo, Rocco Spagnuolo, Maria Diana Naturale, Adele Emanuela De Francesco, Caterina De Sarro, Rossella Romeo, Rita Citraro, Patrizia Doldo, Ludovico Abenavoli, Luca Gallelli, Francesco Luzza, Antonio Leo, Giovambattista De Sarro

**Affiliations:** 1 Department of Health Sciences, Magna Graecia University of Catanzaro, Calabria, 88100 Catanzaro, Italy; 2 System and Applied Pharmacology@University Magna Grecia (FAS@UMG) Research Center, Science of Health Department, School of Medicine, Magna Graecia University of Catanzaro, 88100 Catanzaro, Italy; 3 UOC Pharmacy, “Mater Domini” Hospital, 88100 Catanzaro, Italy; 4 Department of Experimental and Clinical Medicine, Magna Graecia University, 88100 Catanzaro, Italy

**Keywords:** inflammatory bowel diseases, biologics, post-marketing surveillance

## Abstract

Background: The use of immune-modifying biological agents has markedly changed the clinical course and the management of Inflammatory bowel diseases (IBDs). Active post-marketing surveillance programs are fundamental to early recognize expected and unexpected adverse events (AEs), representing a powerful tool to better determine the safety profiles of biologics in a real-world setting. Methods: This study aimed to identify the occurrence of AEs and therapeutic failures linked to biological drugs used in gastroenterology units during a prospective pharmacovigilance program in Southern Italy. Patients affected by IBDs and treated with a biologic agent, from 1 January 2019, to 31 December 2021 (study period) in three gastroenterology units were enrolled. Results: Overall, 358 patients with a diagnosis of active Crohn’s disease or ulcerative colitis satisfying inclusion criteria have been enrolled. Infliximab (IFX) was the most administered drug at the index date (214; 59.8%), followed by Adalimumab (ADA; 89; 24.9%), Golimumab (GOL; 37; 10.3%), Vedolizumab (VDZ; 17; 4.7%) and Ustekimumab (UST; 1; 0.3%). Seventy-three patients (20.4%) experienced at least one AE, while 62 patients (17.3%) had therapeutic ineffectiveness. No serious AEs were reported in the follow-up period in the enrolled patients. AEs have been described with IFX (50/214; *p* = 0.47), GOL (7/37; *p* = 0.78), ADA (13/89; *p* = 0.18), and VDZ (3/17; *p* = 0.52), no AEs have been noticed with UST (0/1). Conclusions: Based on the low rate of AEs observed and withdrawal from treatment, our data seem to corroborate the favorable beneficial/risk profile of biologics for IBDs.

## 1. Introduction

Inflammatory bowel diseases (IBDs) are characterized by recurring episodes of inflammation of the gastrointestinal (GI) tract produced by an aberrant immune response to gut microflora. IBDs embrace two idiopathic intestinal diseases: Crohn’s disease (CD) and ulcerative colitis (UC), which are depicted by a relapsing and remitting course [1,2]. It has been estimated that approximately 2.5 million residents in Europe suffer from IBDs, with considerable costs for healthcare systems [3]. To date, the pathogenesis of IBDs still remains unclear; however, several studies suggest that the development and progression of IBDs are linked to genetics, gut microflora, and dietary habits [4,5]. This intricate interplay of genetic, microbial, and environmental factors leads to chronic activation of the mucosal immune and non-immune response, causing an active inflammation and tissue injury. The mucosal immune system is the main effector of intestinal inflammation and damage, with cytokines having a pivotal role in regulating inflammation [6]. By virtue of this, inflammation seems to be a reasonable target for IBDs therapy using specific inhibitors, which either target inflammatory cytokines or increase the regulatory role of immunosuppressive cytokines [7]. A stepwise treatment is commonly used in the management of IBDs: the immune-modifying agents are added to the regimen when aminosalicylates, corticosteroids (CS), and immunomodulators, such as thiopurines, methotrexate, and cyclosporine A, fail to provide an adequate response [2,8]. Recently, substantial advances in the treatment of IBDs have been achieved after the introduction, in the pharmaceutical market, of several biological agents [9,10]. The immune-modifying biological agents comprise monoclonal antibodies anti-TNF-a and their related biosimilars. In detail, Infliximab (IFX), a chimeric monoclonal IgG1 anti-TNF antibody, and the humanized anti-TNF antibody Adalimumab (ADA) were approved both for CD and UC, whereas the humanized anti-TNF antibody Golimumab (GOL) was only approved for UC [9,11,12,13]. In addition to anti-TNF-α antibodies, biological treatment for IBDs includes Vedolizumab (VDZ), a gut-specific monoclonal antibody that binds to α_4_β_7_ integrins that selectively blocks gut-lymphocyte trafficking and Ustekinumab (UST), a fully humanized IgG1 monoclonal antibody directed toward the p40 subunit of interleukin (IL)-12 and IL23. VDZ was licensed for the treatment of both moderate to severe CD and UC, whereas UST was approved for the management of CD [14,15]. Recently, Tofacitinib, an orally administered small molecule that inhibits the Janus kinase enzyme, was approved for UC [10]. Luckily, the use of these drugs has markedly changed the clinical course and management of IBDs; in fact, they are often effective in treating active disease, inducing and maintaining long-term symptomatic remission in most of these patients [6]. Despite several clinical benefits, biological therapies are not effective in all patients, and many of them lose drug response over time. It has been assessed that in a patient treated with anti-TNF-a almost 30% do not reach clinical improvement (primary failure) and up to 40% develop acquired drug resistance (secondary failure). In addition, unpredictable adverse events (AEs) can occur after treatment with biologics, which are difficult to recognize in pre-marketing clinical trials [16]. In such cases, a switch from one biologic to another provides a valuable clinical solution [17,18]. By virtue of this, clinicians have to be aware of the risk/benefit profile of these agents [19]. Pharmacovigilance studies may offer data on efficacy, and tolerability, also showing potential predictors of ineffectiveness, AEs, and/or severe AEs (SAEs) insurgence [20]. The aim of this project was to detect the occurrence of AEs and therapeutic failures linked to biological drugs used in gastroenterology units during a prospective pharmacovigilance program in Southern Italy, which lasted from 1 January 2019 to 31 December 2021.

## 2. Materials and Methods

### 2.1. Study Design and Data Collection

The Calabria Biologics Pharmacovigilance Program (CBPP) is a multicenter pharmacovigilance project born in 2016 with the aim of improving the monitoring of the safety of biologics in clinical practice [21]. This prospective project was conducted between 1 January 2019, to 31 December 2021 (study period) to assess the safety of biologics treatment in three gastroenterology tertiary units. A monitor, specialist in clinical pharmacology, and/or who received detailed training in pharmacovigilance was allocated for each gastroenterology hospital ward. All consecutive patients undergoing treatment with one biologic drug at three tertiary centers in Calabria Region (Azienda Ospedaliera “Mater Domini”, Catanzaro, Italy; Azienda Ospedaliera Provinciale “San Giovanni di Dio”, Crotone, Italy; Azienda Sanitaria Provinciale (ASP), Cosenza, Italy) were screened for study eligibility. All the consecutive patients that met the following inclusion criteria were enrolled: (1) Age ≥ 18 years; (2) affected by moderate-severe IBD (CD or UC) based on established clinical, endoscopic, radiological, and histological criteria [22], with Harvey Bradshaw Index (HBI) > 8 for CD [23] and Mayo Score (MS) > 6 for UC [24], respectively; and (3) treatment with one biologic drug as per scheduled protocols defined in the respective key registration trial (both monotherapy and co-administration with non-biologic therapy) [25].

Demographic and clinical data from each enrolled patient, such as birth date, sex, diagnosis, duration of disease, first biologic therapy date, current and/or prior biologic therapies, concomitant treatments, reason for discontinuation or switch/swap to another biologic agent, and AEs onset were collected and stored into an ad hoc developed database. A patient-encrypted code was used to guarantee anonymity. Patients with no previous therapeutic exposure to biologics for IBDs were considered bionaïve. The date of the first biologic prescription during the study period represented the “index period” for each patient. Patients were considered to have discontinued therapy whether the biologic drug was withdrawn at the first visit after three months of follow-up.

The reasons for treatment discontinuation were classified as therapeutic inefficacy or occurrence of AEs. AEs were obtained through active reporting and phone calls, as previously reported by Palleria et al., 2018 [21]. For each AE collected, the investigator (physician or pharmacist) recorded a detailed report of the recognized AE, such as the onset time and recovery, seriousness, and outcome. The AE was further confirmed by outpatient clinical assessment. Subsequently, AE was codified by the Medical Dictionary for Regulatory Activities (MedDRA) Preferred Term (PT) and System Organ Class (SOC) levels. An AE was definite as serious (SAEs) if it was life-threatening or fatal, needed hospitalization (or prolonged existing hospitalization), resulted in persistent or significant disability or in a congenital anomaly/birth defect or was another medically critical condition (European Medicines Agency, 2017). Drugs’ SmPC and EudraVigilance have been checked to evaluate previous reports. Furthermore, the estimation of the probability that a biologic drug caused an AE was performed by the Naranjo Adverse Probability Scale, as previously described [26,27]. Subsequently, AEs were reviewed by a pharmacist and registered to the National Pharmacovigilance Center. The study protocol was approved by the local Ethics Committee (Comitato Etico Regionale Calabria, Italy), protocol number 278/2015. All procedures were carried out in agreement with the 1964 Declaration of Helsinki and its later amendments.

### 2.2. Data Analysis

Basal and demographic characteristics of the enrolled patients were evaluated at the index data, using descriptive statistical analyses. Continuous data are expressed as mean ± standard deviation (SD) or median (25–75 percentile) as appropriate, whereas ordinal data are expressed as number (percentage). The Fisher’s exact test or the Pearson chi-squared test for qualitative variables were used to compare data, as previously reported [26]. *p* < 0.05 was considered significant. All statistical procedures were accomplished using IBM SPSS Statistical Software, Version 26.0 (Chicago, IL, USA).

## 3. Results

### 3.1. General Characteristics of the Population Enrolled

Overall, 358 patients (147 females vs 211 males; mean age: 42.8 ± 14.2 years) with a diagnosis of active CD (163; 45.5%) or UC (195; 54.5%) started treatment with a biologic drug have been enrolled. Demographic and clinical data of the patients are summarized in Table 1. 

IFX was the most administered drug at the index date (214; 59.8%), followed by ADA (89; 24.9%), GOL (37; 10.3%), VDZ (17; 4.7%), and UST (1; 0.3%). Data divided per drug are displayed in Table 2. 

Moreover, 104 patients (29%) received concomitant treatment with 1 or more immunomodulatory drugs, non-steroidal anti-inflammatory drugs (NSAIDs), or corticosteroids (CCS). At the index date, 265 (74%) patients were bionaïve to biologic therapy, whereas the remaining 93 (26%) patients were already on treatment with biologics (number of previous biologics range: 1–2; mean (±SD) treatment duration at study entry was 31.9 months ± 24.5). Mean age of patients at first administration of biologics for IBDs was 38.4 (range 16–66; SD ± 14.5). 

Of the 107 total therapies switching in our cohort, 33 (30.8%) were caused by AEs, and the remaining 74 (69.1%) by inefficacy. Concerning switching caused by inefficacy at first biologic, our data showed that 37 (50.7%) of these were referred to IFX, while to a lesser extent the remaining biologics were: 19 (26.0%) ADA, 8 (11%) GOL, and 8 (11%) VDZ 1 (1.4%) UST. On a total of 59 swaps of therapies identified, the most frequently were from IFX to VDZ (25; 42.4%), followed by ADA-VDZ (11; 18.6%), GOL-VDZ (7; 11.9%), VDZ-UST (5; 8.5%), IFX-UST (3; 5.1%), ADA-UST (2; 3.4%), VDZ-GOL (2; 3.4%). Moreover, on a total of 107 switches of therapies within the class of anti-TNFα, the most frequently observed concerns IFX-ADA (25; 52.1%), followed by ADA-IFX (13; 25.0%), GOL-IFX (5; 10.4%), IFX-GOL (4; 8.3%) and to a lesser extent ADA-GOL, VDZ-IFX and UST-VDZ (1; 2.1%). Data shown in Table 3 refer to switches/swaps that occurred during the study period for AEs or treatment ineffectiveness. 

### 3.2. Adverse Events and/or Inefficacy to Treatment

**Figure 1:**
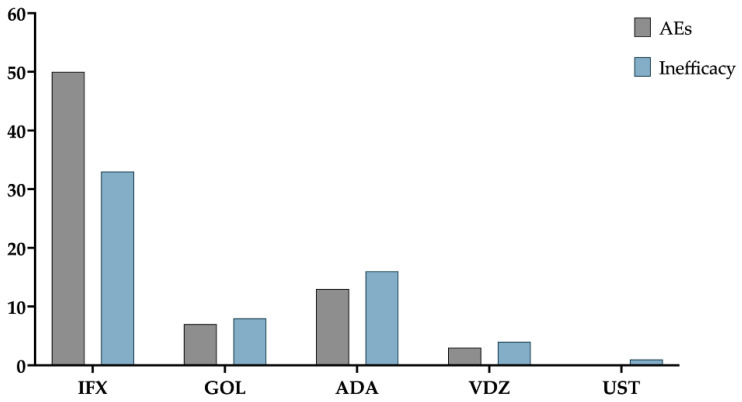
Patients with adverse events (AEs) and inefficacy. IFX: infliximab; ADA: adalimumab; GOL: golimumab; VDZ: vedolizumab; UST: ustekinumab.

During the study period, 223 patients (62.3%) did not report inefficacy or AEs, whereas 73 patients (20.4%) experienced at least one AE, and 62 patients (17.3%) had inefficacy to treatment. AEs occurred in patients as follows: 50 with IFX (50/214; *p* = 0.47), 7 with GOL (7/37; *p* = 0.78), 13 with ADA (13/89; *p* = 0.18), and 3 with VDZ (3/17; *p* = 0.52); no AEs have been noticed with UST (0/1) (Figure 1).

We calculated the AEs incidence rate for each drug by comparing the number of AEs that occurred for each biologic to the number of treatments for that biologic drug. Concerning severity, we found all AEs of mild /moderate grade, whereas no SAEs have been reported in the follow-up period in the enrolled patients. Naranjo probability scale recognized a probable association (Naranjo Score value 7–9) for all AEs observed. According to MedDRA^®^ SOC classification, the most frequently described AEs were general disorders and administration site conditions, most of them related to IFX infusion, followed by skin and subcutaneous tissue disorders and cardiac and nervous system disorders. All the AEs observed were expected, in agreement with as already reported in the summary of product characteristics (SPCs, see Table S1).

Regarding infection, we reported one case of herpes zoster during treatment with GOL and a case of candidiasis correlated to ADA therapy. Furthermore, we reported six cases of AEs related to immune system disorders (hypersensitivity), most of them due to IFX treatment. Surprisingly, we have documented an unexpected case, not reported in the SPCs, of pancreatitis after GOL administration. Specifically, it was a young 36-year-old non-smoker man with UC being treated with GOL, with no comorbidities or concomitant treatment; therefore, no risk factors or conditions predisposing the onset of pancreatitis. Statistical difference was detected in terms of AEs frequency between bionaïve and previously biologically exposed patients (n = 52; 71.2 % vs. n = 21; 28.8%, *p* = 0.03); at odds, bionaïve patients did not display a significant therapeutic ineffectiveness in comparison to previously biologically exposed patients (n = 35; 56.5% vs. n = 27; 43.5%, respectively, *p* = 0.18). Moreover, we observed therapeutic failure with IFX (33/214; *p* = 0.67), GOL (8/37; *p* = 0.98), ADA (16/89; *p* = 0.13), VDZ (4/17; *p* = 0.41) and UST (1/1; *p* = 0.6). There was no significant difference in the number of AEs or inefficacy to treatment between the CD and UC groups. The AEs for each drug, categorized according to the MedDRA dictionary, are detailed in Table 4.

## 4. Discussion

The introduction in clinical practice of biological drugs is considered one of the milestones of modern medicine. Over the past two decades, based on their efficacy and safety, these drugs have profoundly revolutionized the management of several immune-mediated diseases, such as IBDs [28]. Anti-TNF therapy, as expected, was the most frequently prescribed in our study population. Its use is well-established in clinical practice, as it has been the mainstay of treatment for moderate-to-severe IBDs for over 20 years [29]. Compared to IFX and ADA, GOL was only approved for UC, and, in fact, it was lesser administered among anti-TNFs. Moreover, we observed an increase over time in VDZ prescription (third position among biologics with 51 patients). Indeed, it has shown effectiveness, especially in moderate-to-severe UC, becoming a good candidate for first-line biologic therapy [30]. Although many medical societies’ guidelines do not express specific recommendations, the principal management algorithms for choosing therapies based on current evidence include the only use of UST in second-line therapy. We found effectively only one patient bionaïve for UST. Therefore, we can assess that the prescription trend defined in this study complies with scientific evidence and recommendations available in the literature [31].

In our study, the occurrence of AEs during biological therapy was assessed in 223 consecutive patients with IBD during a two-year period, reporting a total of 73 AEs, data consistent with the SPCs. As regards safety, all the AEs identified in this study are known and consistent with those reported in different published studies [32,33]. According to the literature [26,34], the most frequently described AEs were general disorders and administration site conditions, in particular correlated to IFX infusion followed by ADA, which are, however, the most prescribed drugs. General disorders and administration site conditions, such as asthenia, administration site reactions, hot flush, and pyrexia, are among the most frequent AEs observed during biological therapy in our patients. According to the literature, they are very widespread: just think that the estimated incidence of infusion reactions to IFX is greater than 5% [35]. Although the pathophysiology of the immunological mechanism is partially unknown, it seems that patients with antibodies to IFX are at an increased risk of infusion reactions; moreover, case reports suggest hypersensitivity to ADA is also associated with ADA antibodies. However, it must be said that delayed hypersensitivity reaction, albeit rarely, may occur; it presents in the shape of arthralgia, fever, and/or rash from 24 h to 14 days of the infusion, and it is typically managed by antihistamines, paracetamol, and corticosteroids. Actually, we recorded the condition of arthralgia (5 by IFX and 2 by ADA) and pyrexia (2 by IFX) beyond 24 h after administration, which could be considered delayed reactions. For instance, one patient treated with IFX experienced tachycardia and hypotension associated with hot flush and chest pain; these latter are commonly classified as general disorders and administration site conditions, but the evaluation of specific cases suggests a higher affinity to vascular disorders and cardiac disorders (respectively for hot flush and chest pain). Although the action of biological drugs on the immune system may predispose to a greater susceptibility to infections, we did not report severe infections in our study. These findings are consistent with a recent meta-analysis, which found no significant association with the risk of developing severe infections [28,36], and another recent meta-analysis [37] confirmed these results by assessing the efficacy and safety of ADA in UC patients. Literature data suggested that exposure to biologics significantly increases the risk of opportunistic infections in patients with IBD [28], and the variables mainly involved seem to be the elderly, comorbidities, malnutrition or total parenteral nutrition, and intestinal surgery [38]. A recent Italian multicentric study reported two cases of fungal pneumonia and two herpes zoster reactivations in patients treated with IBD treated with biologics [39]. We also reported one case of Herpes Zoster infection during treatment with GOL and one case of candidiasis correlated to ADA therapy. Furthermore, we reported statistically significant differences in AEs between bionaive and non-naive patients (n = 52; 71.2 % vs. n = 21; 28.8%, *p* = 0.02); this could be due to a higher incidence of some AEs between one month and one year after starting biological therapy. In addition, we documented that bionaïve patients did not display a significant therapeutic ineffectiveness in comparison to previously biologically exposed patients (n = 35; 56.5% vs. n = 27; 43.5%, respectively, *p* = 0.18). 

As stated in other studies, absence or loss of efficacy remains the leading cause of switching in clinical practice [26,40]. Indeed, all cases of inefficacy recorded resulted in switching therapies, while a lesser extent percentage of AEs led to the interruption of treatment. First of all, it would be necessary to distinguish between primary non-response (PNR) and secondary loss of response (SLR). In the former case, risk factors for inefficacy can be attributable to characteristics of the patient (smoking, obesity) or disease (for example, longstanding disease). Conversely, SLR occurs when a patient who was in remission on treatment develops symptoms that are proven to be referred to active IBDs. This assessment of inefficacy becomes even more important in the field of pharmacovigilance. Indeed, when patients experience a lack of efficacy, which seems to be related to the drug, the clinician must submit the report to the National Network of Pharmacovigilance (Rete Nazionale di Farmacovigilanza—RNF). 

## 5. Conclusions

Considering the increasing use of biologics in IBDs, as can be seen from the amount of bionaïve patients identified, pharmacovigilance activities play an important role in significantly improving the detection of AEs in clinical practice. In comparison to previous data examined in a prospective study conducted in the same Centers between 2017–2018, we detected a decrease in the rate of AEs reported and a lack of SAEs experienced by patients. Results from this prospective analysis confirm the success of CBPP and other previous pharmacovigilance projects started in our Region with the aim to provide additional information about safety and effectiveness applicable at the stage of prescription. Indeed, this study highlighted greater attention of clinicians in the choice of biologics prescribed and, as a result, a decrease of AEs, especially SAEs, in patients who switched therapies. Conversely, attention to reasons for inefficacy remains an issue on which to focus to enhance the quality of pharmacological treatment: a lack of efficacy related to the drug must be reported in ways that provide a further tool for screening eligible patients for treatment with a specific biologic, consequently reducing con number of switching. 

## Figures and Tables

**Table 1:** Characteristics of the study cohort.

	Overall Patients (n = 358)
Age, years	
Mean (±SD)	42.8 (±14.2)
Range (minimum–maximum)	18–84
Median age (IQ range)	42.0 (31–53)
Sex	
Female, n (%)	147 (41.1)
Male, n (%)	211 (58.9)
Age at first biologic therapy, years	
Mean (±SD)	38.4 (±14.5)
Range (minimum–maximum)	16–66
Median age (IQ range)	37.5 (26–49)
Diagnosis	
Crohn’s diseas, n (%)	163 (45.5)
Ulcerative colitis, n (%)	195 (54.5)
First biologic therapy	
IFX, n (%)	214 (59.8)
GOL, n (%)	37 (10.3)
ADA, n (%)	89 (24,9)
VDZ, n (%)	17 (4.7)
UST, n (%)	1 (0.3)
Concurrent treatments	
MTX, n (%)	2 (0.6)
5-ASA, n (%)	18 (5)
AZA, n (%)	18 (5)
CCS, n (%)	36 (10.1)
NSAIDs, n (%)	30 (8)
AEs/Inefficacy to first biologic agent	
AEs, n (%)	73 (20.4)
Inefficacy, n (%)	62 (17.3)
Switching	
Switch patients, n (%)	107 (29.9)
Swap patients, n (%)	59 (16.4)

Abbreviations: AEs: adverse events; ADA: adalimumab; AZA: azathioprine; CCS: corticosteroids; GOL: golimumab; IFX: infliximab; UST: ustekinumab; VDZ: vedolizumab; MTX: methotrexate; 5-ASA: *mesalazine*; NSAIDs: nonsteroidal anti-inflammatory drugs.

**Table 2:** Characteristics of the study cohort per drugs in first-line biologic therapy.

	IFX	GOL	ADA	VDZ	UST
	(n = 214)	(n = 37)	(n = 89)	(n = 17)	(n = 1)
Age, years					
Mean (±SD)	42.5 (±14.1)	42.6 (±13,3)	42.0 (±14.0)	52.5 (±15.5)	21
Range (minimum–maximum)	(18–84)	(20–72)	(21–71)	(29–84)	21
Median age (IQ range)	42 (30–53)	41 (34–50)	38 (31–53.5)	51 (44–63)	21
Age at first biologic therapy, years					
Mean (±SD)	37.8 (±14.3)	40.2 (±13.5)	37.3 (±14.3)	49.7 (±16.2)	19
Range (minimum–maximum)	(12–80)	(19–70)	(16–67)	(22–80)	19
Median age (IQ range)	38 (25–48.25)	40 (31.5–47.5)	34 (26–49)	49 (36–60.5)	19
Sex					
Female, n (%)	86 (40.2)	15 (40.2)	39 (43.8)	6 (35.3)	1 (100)
Male, n (%)	128 (59.8)	22 (59.5)	50 (56.2)	11 (64.7)	-
Diagnosis					
Crohn’s disease, n (%)	89 (41.6)	1 (2.7)	67 (75.3)	5 (29.4)	1 (100)
Ulcerative colitis, n (%)	125 (58.4)	36 (97.3)	22 (24.7)	12 (70.6)	-
AEs/Inefficacy					
AEs, n (%)	50 (68.5)	7 (9.6)	13 (17.8)	3 (4.1)	-
Inefficacy, n (%)	33 (53.2)	8 (12.9)	16 (25.8)	4 (6.5)	1 (1.6)

Abbreviations: ADA: adalimumab; AEs: adverse events; VDZ: vedolizumab; GOL: golimumab; IFX: infliximab; UST: ustekinumab.

**Table 3:** Details on first switches/swaps biologic therapies and/or second switches/swaps therapies (in brackets) related to inefficacy/AEs.

Switch/Swap to					
Switch/Swap From	IFX	ADA	GOL	VDZ	UST
IFX		25	4	20 (5)	3
ADA	13		(1)	9 (2)	2
GOL	5	-		7	(1)
VDZ	1	(1)	1 (1)		2 (3)
UST	-	-	-	1	

Abbreviations: ADA: adalimumab; AEs: adverse events; VDZ: vedolizumab; GOL: golimumab; IFX: infliximab; UST: ustekinumab.

**Table 4:** SOC-general disorders and administration site conditions.

	IFX	GOL	ADA	VDZ	UST	Total
General disorders and administration site conditions	18	2	3			23
Asthenia	10		1			11
Administration site reactions	2	1	1			4
Hot flush	2	1	1			4
Pyrexia	2					2
Pallor	1					1
Chest pain	1					1
Cardiac disorders	4	1	1			6
Tachycardia	2		1			3
Hypotension	1					1
Presyncope		1				1
Neurocardiogenic syncope (vasovagal syncope)	1					1
Skin and subcutaneous tissue disorders	6	1	2	2		11
Pruritus	4	1		1		6
Urticaria	2		1	1		4
Hidradenitis			1			1
Nervous system disorders	6					6
Somnolence	1					1
Confusional state	1					1
Headache	4					4
Infections and infestations		2	2			4
Candidiasis infection			2			2
Herpes zoster		2				2
Respiratory, thoracic, and mediastinal disorders	3		2			5
Tonsillitis	1					1
Dyspnoea	1		2			3
Nasopharyngitis	1					1
Gastrointestinal disorders	2	1		1		4
Nausea	2			1		3
Pancreatitis		1				1
Immune system disorders	5		1			6
Hypersensitivity	5		1			6
Musculoskeletal and connective tissue disorders	5		2			7
Arthralgia	5		2			7
Blood and lymphatic system disorders	1					1
Leucocytosis	1					1
Total	50	7	13	3	0	73

## Data Availability

Not applicable.

## Supplementary Materials

The following supporting information can be downloaded at: https://www.mdpi.com/article/10.3390/pharmaceutics14112449/s1, Table S1: Link to Summary of Product Characteristics (SPCs) for the biologics considered.
