# Supplementary material for: Biologics for Inflammatory Bowel Disease in Clinical Practice: A Calabria (Southern Italy) Prospective Pharmacovigilance Study
Source: Pharmaceutics. 2022 Nov 13;14(11):2449. doi: 10.3390/pharmaceutics14112449 (PMC9696291; doi:10.3390/pharmaceutics14112449)
Supplement: Supplementary file 1 [file pharmaceutics-14-02449-s001.zip › pharmaceutics-1976671-supplementary.pdf]

Table S1: Link to Summary of Product Characteristics (SPCs) for the biologics considered.

Biologic drug	Link
Infliximab (IFX)	https://www.ema.europa.eu/en/documents/product-information/remicade-epar-product-information_en.pdf
Adalimumab (ADA)	https://www.ema.europa.eu/en/documents/product-information/humira-epar-product-information_en.pdf
Golimumab (GOL)	https://www.ema.europa.eu/en/documents/product-information/simponi-epar-product-information_en.pdf
Vedolizumab (VDZ)	https://www.ema.europa.eu/en/documents/product-information/entyvio-epar-product-information_en.pdf
Ustekinumab (UST)	https://www.ema.europa.eu/en/documents/product-information/stelara-epar-product-information_en.pdf
